# Stable Internal Reference Genes for the Normalization of Real-Time PCR in Different Sweetpotato Cultivars Subjected to Abiotic Stress Conditions

**DOI:** 10.1371/journal.pone.0051502

**Published:** 2012-12-12

**Authors:** Sung-Chul Park, Yun-Hee Kim, Chang Yoon Ji, Seyeon Park, Jae cheol Jeong, Haeng-Soon Lee, Sang-Soo Kwak

**Affiliations:** 1 Environmental Biotechnology Research Center, Korea Research Institute of Bioscience and Biotechnology, Daejeon, Republic of Korea; 2 Department of Green Chemistry and Environmental Biotechnology, University of Science and Technology, Daejeon, Republic of Korea; Virginia Tech, United States of America

## Abstract

Reverse transcription quantitative real-time PCR (RT-qPCR) has become one of the most widely used methods for gene expression analysis, but its successful application depends on the stability of suitable reference genes used for data normalization. In plant studies, the choice and optimal number of reference genes must be experimentally determined for the specific conditions, plant species, and cultivars. In this study, ten candidate reference genes of sweetpotato (*Ipomoea batatas*) were isolated and the stability of their expression was analyzed using two algorithms, geNorm and NormFinder. The samples consisted of tissues from four sweetpotato cultivars subjected to four different environmental stress treatments, i.e., cold, drought, salt and oxidative stress. The results showed that, for sweetpotato, individual reference genes or combinations thereof should be selected for use in data normalization depending on the experimental conditions and the particular cultivar. In general, the genes *ARF*, *UBI, COX, GAP* and *RPL* were validated as the most suitable reference gene set for every cultivar across total tested samples. Interestingly, the genes *ACT* and *TUB*, although widely used, were not the most suitable reference genes in different sweetpotato sample sets. Taken together, these results provide guidelines for reference gene(s) selection under different experimental conditions. In addition, they serve as a foundation for the more accurate and widespread use of RT-qPCR in various sweetpotato cultivars.

## Introduction

To analyze the expression profile of genes of interest, comparative measurements such as microarray, Northern blot, reverse transcription-PCR (RT-PCR), and real-time RT-PCR (RT-qPCR) are frequently used [1]. Among these, RT-qPCR is the simplest, most sensitive, most precise, and cost-effective quantitative method allowing the detection of both low-abundance mRNAs and slight variations in gene expression. Thus, RT-qPCR has become the preferred approach to the validation of high-throughput or microarray results and the quantitation of gene expression [1], [2]. However, there are substantial variations in RNA stability, quantity, and purity that in turn influence the efficiency of RT-qPCRs [3]. In fact, several reports have demonstrated that there is no gene set able to fulfill all the requirements of every experimental condition, and that improper reference-gene selection could yield inaccurate results [1], [4], [5]. Therefore, the selection of a reliable internal control is essential, as is the use of standardized experimental conditions [6]–[10]. Current experimental convention includes the use of a single gene for normalization; however, this may lead to relatively large errors in a significant proportion of the samples [9], [11]. Alternatively, multiple internal control genes will ensure accurate normalization of the data [9], [12], [13]. This implies that, prior to their use in RT-qPCR normalization, potential reference genes must be systematically evaluated for their stability under the applied experimental conditions. Several algorithms, such as geNorm [14], are currently available as part of qBase^Plus^ [15], NormFinder [16] and BestKeeper [17]. They have been developed to validate for a given set of experimental conditions the most stable reference gene(s) from a panel of potential genes or candidate genes.

Gene expression analyses under a wide range of experimental conditions have relied on the use of traditional housekeeping genes such as actin (*ACT*), tubulin (*TUB*), glyceraldehyde-3-phosphate dehydrogenase (*GAP*), elongation factor-1α alpha (*EF1α*), and 18S rRNA, for normalization of the data. However, in many cases, the transcripts expressed from these genes are unstable, such that variations in the respective expression levels can lead to a misinterpretation of the results. Recently, statistical algorithms have been used to identify the best reference genes for RT-qPCR data normalization in a given set of biological samples. These algorithms have been applied to assess the expression stability of numerous candidate reference genes across a variety of tissues, organs, developmental stages, and stress conditions in various plant species, such as *Arabidopsis* [6], [18], tobacco [19], rice [20], tomato [7], potato [21], poplar [13], cotton [4], cucumber [12], banana [9], grapevine [22], soybean [23], coffee [24], *Brachiaria* grass [25], and petunia [26].

Sweetpotato [*Ipomoea batatas* (L.) Lam] is, together with cassava, one of the most important commercial and nutritional root crops in Asia. It is used not only as a major food source but also as an important industrial raw material for animal feed, alcohol production and antioxidant pigment syntheses, including anthocyanins and carotenoids [27], [28]. Recently, new varieties of colored sweetpotatoes, including yellow-fleshed and purple-fleshed varieties with higher carotenoid and anthocyanin contents and desirable nutritional and physiological properties, have been introduced.

**Table 1:** Primer sequences of selected candidate reference genes, primers, and amplicon characteristics.

Name	GenBank accession number	Primer sequence (forward/reverse)	Amplicon length	Primer efficiency	R2
ACT	EU250003.1	GTTATGGTTGGGATGGGACA	199	95.8±3.1	0.995
		GTGCCTCGGTAAGAAGGACA			
ARF	JX177359	CTTTGCCAAGAAGGAGATGC	185	100±0.5	0.999
		TCTTGTCCTGACCACCAACA			
COX	S73602.1	ACTGGAACAGCCAGAGGAGA	159	99.2±1.7	0.998
		ATGCAATCTTCCATGGGTTC			
CYC	EF192427.1	GGATCGAAGTTCAAGGACGA	183	93.8±1.3	0.999
		CTTCACCACGTCCAAACCTT			
GAP	JX177362	ATACTGTGCACGGACAATGG	124	99.8±0.7	0.999
		TCAGCCCATGGAATCTCTTC			
H2B1	JX177361	GTGCCGGAGACAAGAAGAAG	110	101.9±2.8	0.998
		CTTGCTGGAGATTCCGATGT			
PLD	JX177360	ATCGGAATCAGCAGTGATGG	144	101.0±3.2	0.991
		ATGATGAGGCAAGCAGTGTG			
RPL	AY596742.1	TTTGACCGAAATGCCCTTAG	160	105.2±7.4	0.997
		TTCTGGTTCACCCCAACATT			
TUB	BM878762.1	TCCAAACCAACCTTGTACCC	198	96.8±3.1	0.995
		CTCGGTACATCAAGCAGCAA			
UBI	JX177358	TCGACAATGTGAAGGCAAAG	209	99.9±3.4	0.996
		CTTGATCTTCTTCGGCTTGG			

From a scientific standpoint, sweetpotato is a valuable resource for studying the developmental and physiological properties of root crops, such as storage root development, sterility, and cross-incompatibility. In RT-PCR and RT-qPCR studies of sweetpotato cultivars exposed to various stress conditions, *ACT* or *TUB* usually serve as reference genes [29]–[31], even though their stability has not been verified. Therefore, we aimed to identify stable reference genes for use in RT-qPCR studies in sweetpotato. To this end, four cultivars of pigmented sweetpotato were exposed to four different stress conditions and the validity of the candidate genes as reference genes was subsequently validated. The identification and validation of these reference genes for use in RT-qPCR normalization will significantly improve the accuracy and reliability of gene expression studies in sweetpotato.

## Materials and Methods

### Plant Materials and Growth Conditions

Four different sweetpotato cultivars, Yulmi (YM, heart-shaped leaves and pale-yellow flesh storage roots), Sinzami (SZM, long spade-like leaves and purple flesh storage roots), Sinhwangmi (SHM, heart-shaped leaves and orange flesh storage roots), and Whitestar (WS, lobed leaves and white flesh storage roots), were placed in a growth chamber at 25±3°C for 3 months (Figure S1). Freshly harvested leaves, petioles, stems, fibrous roots (<5 mm in diameter), and pencil roots (<15 mm in diameter) were sampled 12 weeks after planting. Mature storage roots (>15 mm in diameter) were obtained from the Bioenergy Crop Research Center, National Institute of Crop Science, Rural Development Administration (RDA), Korea. The tissues of all plant materials were immediately frozen in liquid nitrogen and stored at −70°C until further use.

**Figure 1:**
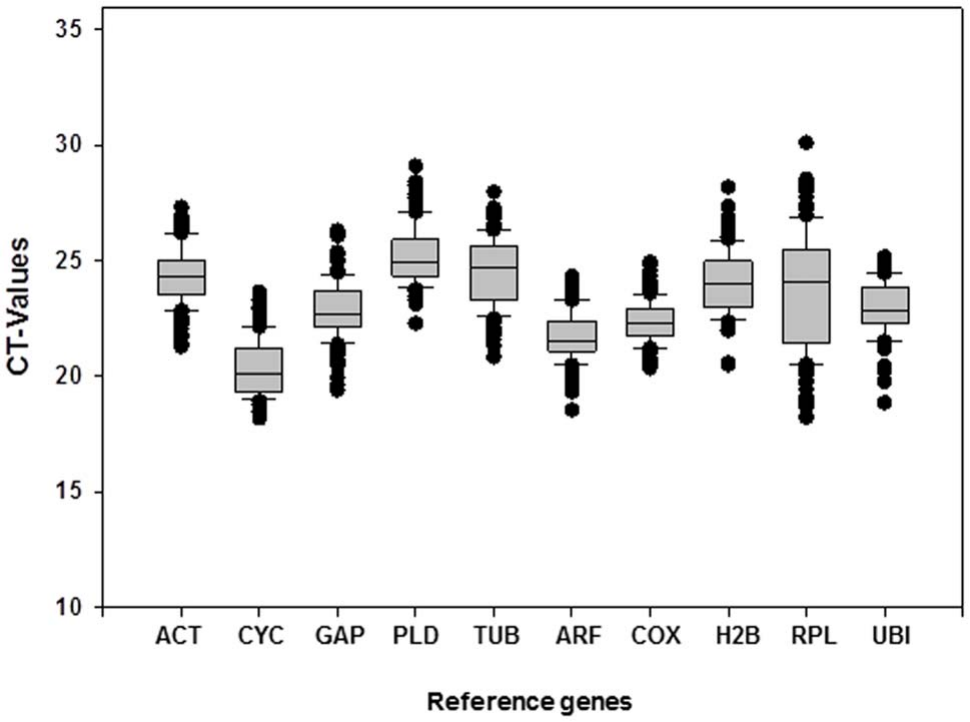
RT-qPCR CT values for the candidate reference genes. Expression data are displayed as CT values for each reference gene in all sweetpotato samples. Maximum and minimum CT values for each gene are shown.

### Stress Treatment

Fully expanded mature leaves from the third and fourth vine tip of each cultivar were detached approximately 2 months after transplantation of a 10 cm-long segment of the upper part of the vine into a pot (Figure S1). For cold stress treatments, the pots were incubated at 4±1°C for 0, 2, 4, 6, 12, 24, 48 and 72 h in the dark. For drought and salt stress treatments, the petioles of detached leaves were soaked in 30% PEG and 250 mM NaCl, respectively, for 0, 2, 4, 6, 12, 24, 48 and 72 h. For oxidative stress treatments, sweetpotato leaves were treated with 400 mM H_2_O_2_ for 0, 2, 4, 6, 12 and 24 h. All treated plant materials were immediately frozen in liquid nitrogen and stored at −70°C until further use.

### RNA Isolation, Quality Control, and cDNA Synthesis

Frozen samples were ground in liquid nitrogen using a mortar and a pestle. Total RNA was exacted with Trizol reagent (Invitrogen, USA) and then treated according to the manufacturer’s instructions with DNase I (Takara, Japan) to remove any traces of genomic DNA. The concentration and purity of the RNA were determined using a Nanodrop 1000 spectrophotometer (Thermo, USA). RNA samples with 260/280 ratio in the range of 1.8–2.1 and a 260/230 ratio in the range of 2.3–2.6 were used for subsequent analyses. The integrity of the RNA samples was also electrophoretically assessed on a 2.0% agarose/formaldehyde gel. Two micrograms of total RNA was reverse-transcribed using the M-MLV cDNA synthesis kit (Clontech, USA) and oligo-dT primers according to the manufacturer’s instructions.

**Figure 2:**
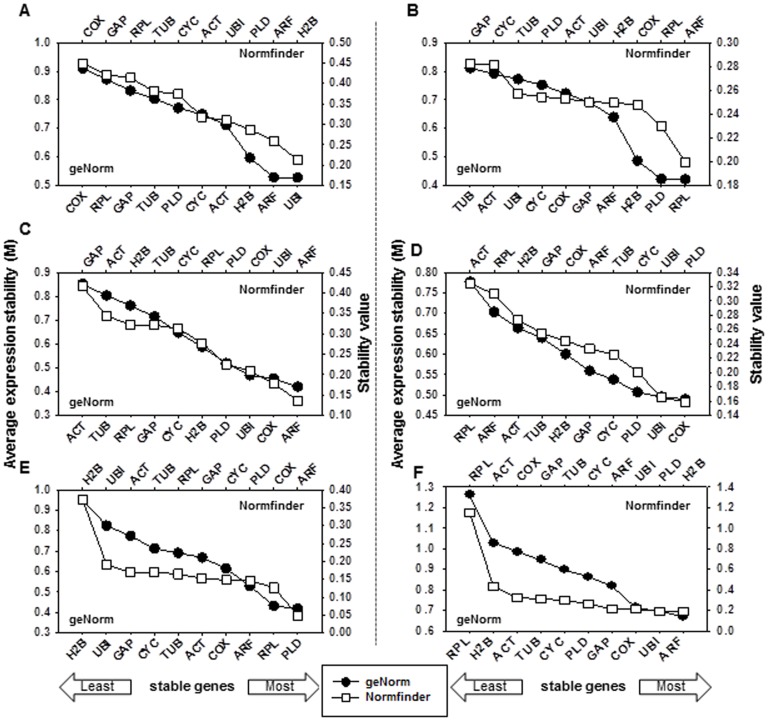
Average expression stability values (*M*) and the stability values in different data sets obtained from the software geNorm and NormFinder, respectively. (A) Cold stress, (B) oxidative stress, (C) salt stress, (D) drought stress, (E) tissues, and (F) total.

### Primer Design and RT-qPCR

The sequences of ten sweetpotato reference genes were obtained from the GenBank database and from the EST database [32]. The primer pairs were designed from these sequences with the Primer3plus program (Table 1) (http://www.bioinformatics.nl/cgi-bin/primer3plus/primer3 plus.cgi). Before RT-qPCR analysis, each primer pair was tested with RT-PCR to determine the size specificity of the amplicon, followed by electrophoresis on a 2% agarose gel and ethidium bromide staining. In addition, target amplicons were sequenced to confirm specificity of the PCR products. The primer specificities were further assessed by melting-curve analysis after amplification in a RT-qPCR study. A standard curve, repeated in three independent plates using a dilution series of the mixed cDNAs obtained from all tested samples as templates. Then we calculated the gene-specific PCR amplification efficiency and correlation coefficient of each gene. The primer sequences and amplicon characteristics, including Tm, length, amplification efficiency with standard deviation, and correlation coefficient, of the ten candidate reference genes are listed in Table 1. RT-qPCR analysis was carried out in 96-well plates with a CFX real-time PCR system and CFX system software (Bio-Rad, USA) using the EvaGreen-based PCR assay. Each reaction (final volume of 20 µl) contained 2 µl diluted cDNAs, 10 µl of EvaGreen PCR Master Mix (Solgent, Korea), and 1 µl of each primer. The PCR conditions were as follows: 95°C for 15 min, followed by 40 cycles of 95°C for 20 s, 60°C for 40 s, and 72°C for 20 s. The melting curves were analyzed at 65–95°C after 40 cycles. Each RT-qPCR was performed in duplicate.

**Table 2:** Optimal number of stability gene(s) estimated by geNorm and Normfinder under abiotic stresses and in the tissue of each cultivar and all cultivars.

		Most stable gene(s)				Most stable gene(s)	
Cultivar	Samples	geNorm	Normfinder	Cultivar	Samples	geNorm	Normfider
YM	Tissue	TUB, ACT	ARF	SHM	Tissue	COX, TUB	ARF
	Cold	UBI, ARF	UBI		Cold	H2B, UBI	ARF
	Oxidative	PLD, RPL	ARF		Oxidative	COX, GAP	ARF
	Salt	UBI, ARF	ARF		Salt	H2B, ARF	ARF
	Drought	GAP, ARF	GAP		Drought	ARF, GAP	UBI
	All stresses	ARF, UBI, COX, CYC	UBI		All stress	ARF, UBI	UBI
SZM	Tissue	PLD, RPL	GAP	WS	Tissue	COX, UBI	ARF
	Cold	UBI, ARF	H2B		Cold	PLD, RPL	H2B
	Oxidative	PLD, RPL, ARF	ARF		Oxidative	ARF, COX	ARF
	Salt	COX, PLD	COX		Salt	ARF COX, UBI	ARF
	Drought	TUB, UBI	GAP		Drought	COX, H2B	PLD
	All stresses	ARF, UBI	ARF		All stress	COX, UBI, H2B, ARF	PLD
		**Most stable gene(s)**					
**Cultivar**	**Samples**	**geNorm**		**NormFinder**		**NormFinder best pair**	
Allcultivars	Tissue	PLD, RPL, ARF, COX		ARF		ARF, COX	
	Cold	UBI, ARF, H2B, ACT		H2B		ACT, ARF	
	Oxidative	RPL, PLD, H2B, ARF		ARF		CYC, GAP	
	Salt	ARF, COX, UBI		ARF		ARF, UBI	
	Drought	COX, UBI, PLD		PLD		CYC, PLD	
	All stresses	UBI, COX, ARF, H2B		H2B		ARF, COX	
	Total	ARF, UBI, COX, GAP, PLD		H2B		PLD, UBI	

### Determination of Reference-gene Expression Stability

The stability of housekeeping-gene expression under different experimental conditions was determined using two statistical algorithms, geNorm and NormFinder, in a stability analysis of 10 candidate reference genes. The geNorm program examines the stability of expression as well as the optimal number of reference genes needed for normalization. It first calculates an expression stability value (*M*) for each gene and then the pairwise variation (*V*) of this gene with the others. The lowest stability value represents the gene with the most stable expression within the gene set examined. All tested genes are ranked according to their stability in the tested samples and the number of reference genes required to obtain an optimal normalization is indicated. NormFinder determines the stability of expression as well as the optimal gene or combination of genes for normalization purposes. It ranks the set of candidate normalization genes according to the stability of their expression in a given sample set under a given experimental design.

**Figure 3:**
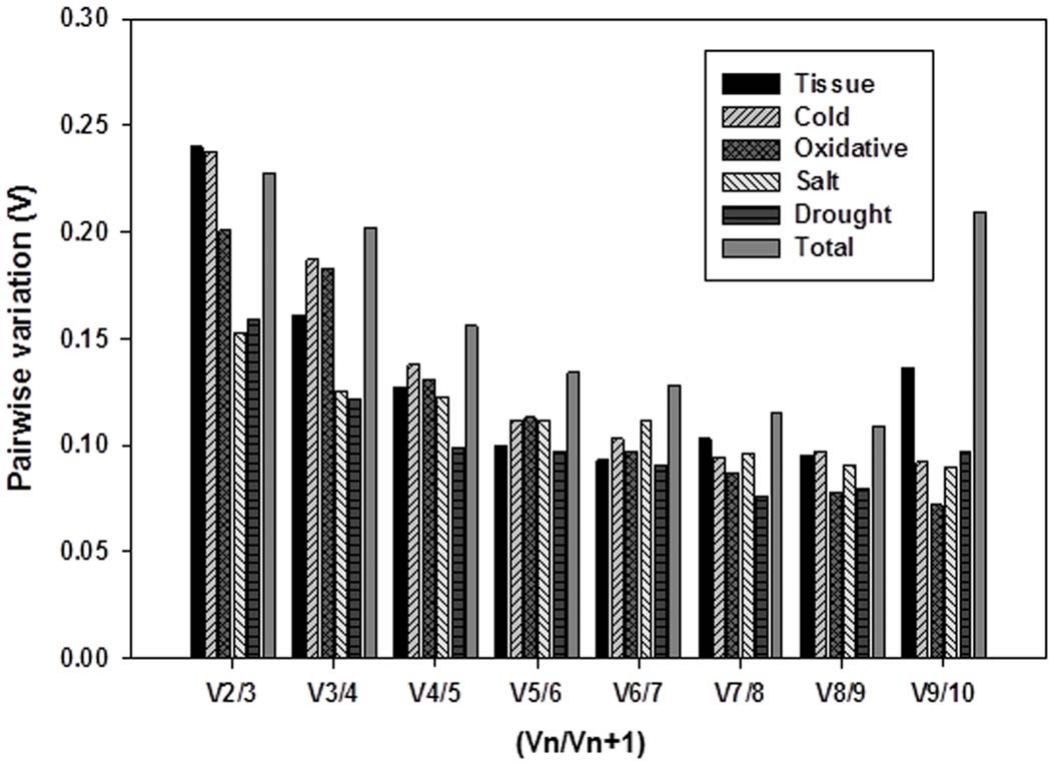
Pairwise variation (*V*) analysis of the candidate reference genes. The geNorm software was used to analyze the pairwise variation (*Vn/Vn +1*) between the normalization factors *NF_n_* and *NF_n+1_* in order to determine the optimal number of reference genes required for RT-qPCR data normalization.

### Normalization of *swpa2* and *IbLEA14*

Expression of the stress-inducible sweetpotato marker genes *IbLEA14* and *swpa2* were quantified during oxidative stress conditions (400 mM H_2_O_2_) using one or two of the most stable reference genes and *TUB*, a gene commonly used as a housekeeping gene in studies of sweetpotato. Primer pairs for *IbLEA14* (forward: GCCCTGGATGTGGCAGTGAA, reverse: GGCAGCTTCTGCCTCTGCTTC) and *swpa2* (forward: GAGTTGAGCTCGAAATCCGTGA, reverse: CCCCCTTTATTTCCAACAAGCA) were used in these gene validation experiments.

## Results

### Isolation of Sweetpotato References Genes and Verification of their Amplicons, Primer Specificity, and PCR Amplification Efficiency

We selected ten candidate reference genes for this study based on their use in prior gene expression experiments. These genes include *β-actin* (*ACT*), *ribosomal protein L* (*RPL*), *glyceraldehyde-3-phosphate dehydrogenase* (*GAP*), *cyclophilin* (*CYC*), *α-tubulin* (*TUB*) [8], [21], [33], *ADP-ribosylation factor* (*ARF*) [34], *histone H2B* (*H2B*) and *ubiquitin extension protein* (*UBI*) [13], and *cytochrome c oxidase subunit Vc* (*COX*) [35]. The expression of *phospholipase D1α* (*PLD*) in castor bean [36], Arabidopsis [37] and rice [38] suggests a maintenance role in plant cells and was thus included in the study.

**Figure 4:**
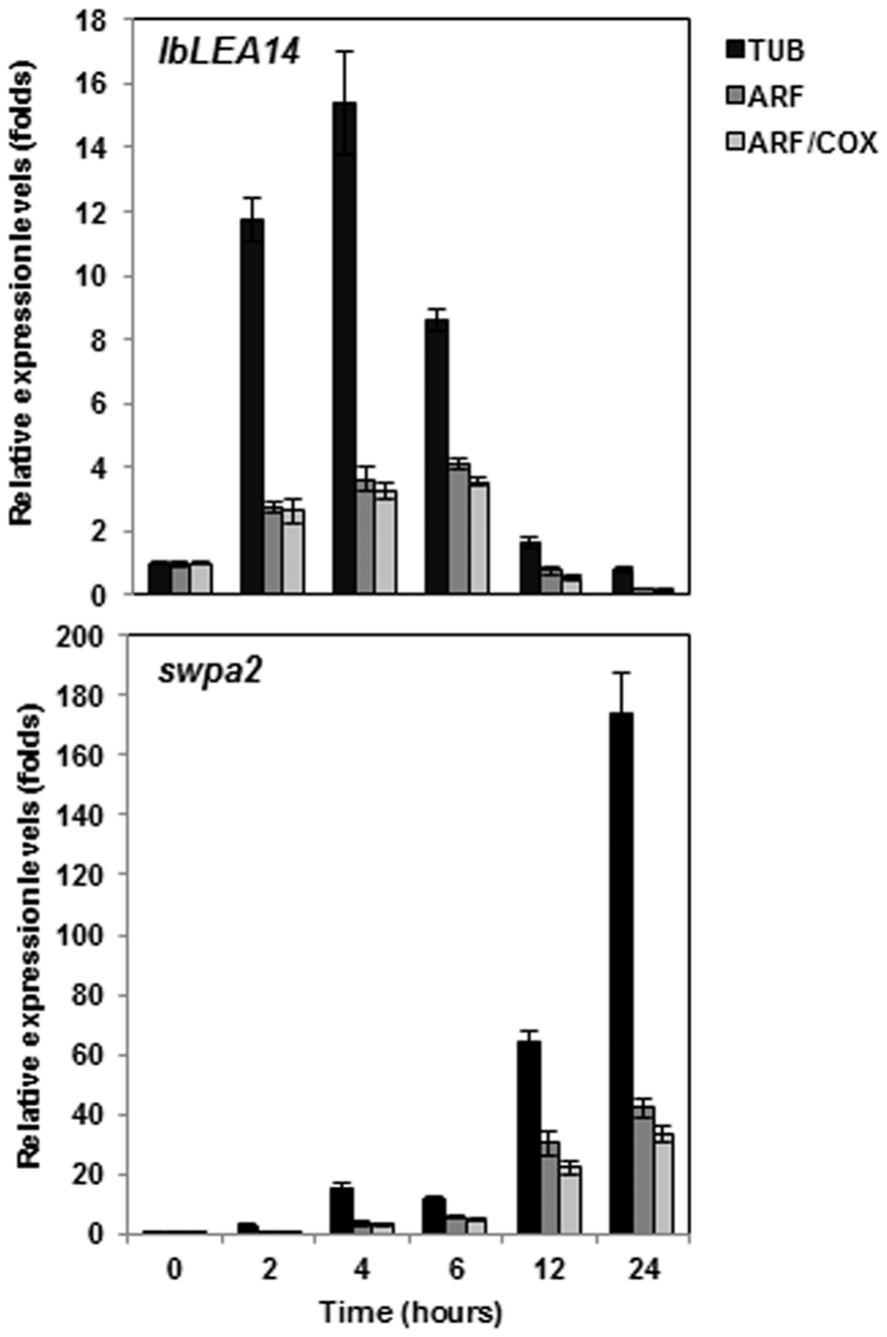
Relative quantification of *IbLEA14* and *swpa2* expression using validated reference genes for normalization under oxidative stress in the White star cultivar. (A) *IbLEA14* and (B) *swpa2.*

Nucleotide sequences for *ACT, CYC, TUB, COX*, and *RPL* were obtained from GenBank database and those for *GAP, PLD, ARF, H2B* and *UBI* were obtained from the sweetpotato EST database [32] (Table 1). Specific amplification for each tested primer pair was confirmed by the presence of a single peak in melting curve analysis proceeding 40 cycles of amplification (Figure S2). Furthermore, each amplicon was cloned and sequenced, matching the predicted target sequence. Sequence analysis of cloned amplicons revealed that all sequenced amplified fragments were identical or nearly identical to the sequences used for primer design, with 1–5 bp mismatched (but the sequences of amino acids were fully identical). The RT-qPCR amplification efficiency for the ten reference genes varied from 93.8% for *CYC* to 105.2% for *RPL*; correlation coefficients ranged between 0.991 (*PLD* and *ARF*) and 0.999 (*CYC* and *GAP*) (Table 1).

### Expression Profiles of the Reference Genes

Analysis of the raw expression levels across all samples identified some degree of variation among the reference genes (Figure 1). The cycle threshold values of the ten reference genes ranged from 20.33 (*CYC*) to 25.23 (*PLD*) in all tested samples. Regarding variations in the expression of individual reference genes, the highest values were obtained for *RPL* (11.97 cycles) and *H2B* (7.37 cycles), and the lowest values for *COX* (4.50 cycles) and *CYC* (4.56 cycles). Thus, none of the selected genes were consistently expressed in all samples, highlighting the importance in sweetpotato studies of identifying a suitable reference gene(s) for use in gene expression normalization under a given set of experimental conditions.

### Expression Stability of Candidate Reference Genes

To find the most stably expressed genes for sweetpotato RT-qPCR normalization, the expression stability of ten candidate genes was assessed using statistical methods. Specifically, two widely applied algorithms, geNorm and NormFinder, were used to rank the stabilities of the ten genes and to determine the number of reference genes necessary for accurate gene expression profiling under the experimental conditions selected for this study.

The results obtained with these algorithms are presented in Figure 2 and summarized in Table S1. Among the ten candidate reference genes examined when all samples were considered, geNorm identified *ARF* (*M* = 0.669) as the most stable and *RPL* (*M* = 1.262) as the least consistently expressed. In contrast, *H2B* and *PLD* (0.192) were determined by NormFinder to be the most stable reference genes, whereas *RPL* (1.152) was again ranked as the most variable (Figure 2F and Table S1). Pairwise variation (*V_n/n+1_*) analysis showed the need for an optimal number of reference genes to obtain a more reliable normalization according to geNorm (*V_n/n+1_*≤0.15), with the genes *ARF*, *UBI*, *COX*, *PLD*, and *GAP* (*V_5/6_* = 0.134) suitable for proper normalization (Figure 3).

*UBI* was the most stable gene in geNorm, while in plants under cold stress, *H2B* was considered by NormFinder as the most stable (Figure 2A and Table S1). A stable combination of two genes according to NormFinder was *ACT/ARF*. In contrast, *COX* was identified as the least stable gene both in geNorm and in NormFinder. Analysis of the pairwise variation revealed that the *UBI/ARF/H2B/ACT* genes (*V4/5* = 0.138) are sufficient for normalizing gene expression (Figure 3). These genes were considered the most variable reference genes in most cultivars according to both algorithms, whereas for the WS cultivar, geNorm identified only *PLD* and *RPL* as reference genes (Table 1, 2 and Figure 2).

During oxidative stress conditions, elicited by 400 mM H_2_O_2_, *RPL* was selected by geNorm as the most stable gene among all reference genes, whereas *ARF*, followed by *RPL*, was the most stable in NormFinder (Figure 3B and Table S1). *TUB* was identified by geNorm as the least stable gene, whereas *GAB* was the most stable in NormFinder. A combination of four reference genes, *RPL/PLD/H2B/ARF*, comprised the optimum and most stable gene set under oxidative stress, according to geNorm (*V4/5* = 0.131). While *ARF* was the most stable gene in every cultivar according to NormFinder (Table S1), geNorm identified different gene sets in cultivars SHM and WS (*COX/GAP* and *ARF/COX*, respectively) (Table 2 and Figure S3).

*ARF* was calculated by both geNorm and NormFinder to be the most stable gene under salt stress, followed by *COX* or *UBI* (Figure 2C). The two-gene combination according to NormFinder comprised *ARF* and *UBI*. Analysis of the pairwise variation revealed *ARF/COX/UBI* (V3/4 = 0.125) as the suitable reference genes in geNorm (Figure 3). *ACT* was the least stable gene in geNorm and *GAB* in NormFinder,. Two cultivars, SHM and SZM, differed in their reference gene sets (*H2B/ARF* and *COX/PLD*, respectively) in geNorm, whereas in other cultivars, the gene set *ARF/COX/UBI* was identified as the most suitable (Table 2 and Figure S3).

Under conditions of drought stress, *COX* was calculated to be the most stable gene in geNorm and *PLD* in NormFinder (Figure 2D). The least stable gene under drought stress was *RPL* in geNorm, while in NormFinder it was *ACT*. For all PEG-treated cultivars, even though *PLD/UBI/COX* constituted the most appropriate reference gene set (*V3/4* = 0.122), the values for each cultivar differed (Figure 3 and Table 2). According to geNorm, in cultivars YM and SHM the optimal combination was *GAP/ARF*, while in cultivars SZM and WS it was *TUB/UBI* and *COX/H2B*, respectively. In NormFinder, the best gene was *GAP* in cultivars YM and SZM, while *UBI/PLD* were the most stable genes in SHM and WS (Table 2, S1).

Analysis of the best reference genes in each experimental subset showed several differences (Table 2). Six different tissues, leaf, petiole, stem, fibrous root, pencil root and storage root, were analyzed. In geNorm, *PLD* was ranked as the most stable reference gene followed by *RPL* and *ARF*, whereas *ARF* was the most stable gene according to NormFinder (Figure 2E). In contrast, *H2B* was identified as the least stable gene by both geNorm and NormFinder. The two-gene combination in NormFinder was *ARF/COX*. Analysis of the pairwise variation revealed that the *PLD/RPL/ARF/COX* genes (V4/5 = 0.127) are sufficient for normalizing gene expression (Figure 3). These genes were considered by both algorithms as the most variable reference genes. *ACT/TUB* (YM), *COX/TUB* (SHM), *COX/UBI* (WS) and *PLD/RPL* (SZM) were identified by geNorm as the most stable gene set, while according to NormFinder, *ARF* ranked highest in terms of stability in every cultivar except SZM (*GAP*) (Figure 2 and Table2, S1). The stable gene sets specific to each cultivar under different stress conditions are presented in Table 2. A comparison of the results obtained with geNorm and NormFinder identified *ARF/UBI/COX/PLD/GAP* as the most stable reference gene combination for all samples and subsets tested in this study, thus supporting its use as reference gene sets for accurate transcript normalization in sweetpotato.

### Reference Gene Validation

To demonstrate the usefulness of the validated candidate reference genes in RT-qPCR, the relative expression levels of two sweetpotato marker genes, *IbLEA14* and *swpa2*, already reported to be stress-inducible [31], [39] were investigated under oxidative stress conditions (400 mM H_2_O_2_), together with one or two of the most stable reference genes, validated by geNorm and NormFinder (Figure 2 and Table S1), as well as *TUB*, a gene commonly used to normalize expression (Figure 4). The results showed that, for normalization, the expression levels of two of the chosen stable reference genes, *ARF/COX*, underwent similar changes, while slight differences were observed when *ARF* was used alone. In response to H_2_O_2_ treatment, *IbLEA14* expression increased progressively for 6 h and then decreased between 12 and 24 h, whereas *swpa2* expression increased gradually until 24 h. Although these changing expression patterns were confirmed when normalized using *TUB*, the expression levels of the examined reference genes suggested a completely different result, in that the two genes were initially expressed at extremely high levels followed, after 6 h of stress treatment, by a sharp decrease. This variation illustrates the adverse consequences of using an unsuitable reference gene for normalization.

## Discussion

In this study, the results identified *ARF, UBI, COX, GAP* and *PLD* as the most stably expressed reference genes in all samples and subsets studied in sweetpotato (Figure 2 and Table 2, S1). This observation reinforces the necessity to assay the stability of expression of candidate genes to select suitable reference genes, allowing a reliable normalization in a specific biological assay. In addition, in sweetpotato cultivars subjected to various abiotic stress conditions, *ARF* and *UBI* were shown to be good candidate reference genes (Table 2). Consistent with our results, Carvalho *et al.* [34] reported that *ARF* and *EF-1α* yielded the most stable transcript accumulation in *Swingle citrumelo* subjected to drought stress. In the adventitious or lateral root development of poplar, the most stable reference gene set consisted of *UBI* and *RPL* [13]. Therefore, present study is the first to identify stable internal reference genes in different sweetpotato cultivars.

There is increasing evidence that a single reference gene cannot be used to accurately normalize RT-qPCR data; rather, a combination of multiple reference genes is needed. However, although increasing the number of reference genes for normalization will improve the accuracy of the analysis, this is expensive as well as time-consuming. It also has been suggested that the number of reference genes that must be analyzed is dependent on the purpose of the study [9], [40]. The use of two stable reference genes provides a valid normalization for most experimental conditions [9]. This was confirmed in the present study, in which for most sample sets, two genes were shown to be sufficient to obtain a more accurate and more reliable normalization than achieved with the use of a single reference gene (Figure 3 and Figure S3). In addition, our results showed that the choice of the best combination of reference genes depends on the experimental conditions. Interestingly, the best reference genes differed for the various samples (Figure 3 and Table 2, S1). For example, in samples obtained from six different tissues, *ACT* and *TUB* ranked as a stable gene set in cultivar YM, whereas in the cultivars SHM, SZM, and WS, the gene sets *COX/TUB*, *PLD/RPL*, and *COX/UBI*, respectively, were better. Under drought stress, *GAP* and *ARF* were the best reference genes in YM and SHM, but when these two cultivars were placed under oxidative stress, *PLD*/*RPL* and *COX*/*GAP* were the most stable genes. Finally, *UBI* was the highest ranked reference gene for all stresses in all cultivars, whereas *UBI* and *H2B* appeared to be the least stable reference genes in all tissues, except those of cultivar WS (*H2B*/*GAP*). These results provide further support for both the need to use a specific set of reference genes and the importance of validating those genes for each experimental condition tested, especially in greatly differing samples. Findings similar to ours have been reported for banana [9], citrus [40], and tomato [7].

For the purpose of experimental data analysis, this study evaluated candidate genes in terms of their expression stability, using statistical methods to rank gene stabilities and to determine the number of reference genes necessary for accurate gene expression profiling under specific experimental conditions. The most widely used algorithms, geNorm, NormFinder or Bestkeeper were selected for the analysis. Some studies in which both geNorm and NormFinder were applied reported minor differences in the gene stability rankings [4], [9], [12], while in studies of citrus and wheat, the differences were substantial [40], [41]. In the present work, the two methods yielded slightly different rankings (Table S1). Because the Bestkeeper algorithm can process 100 samples, whereas our study was based on 144 samples, ruling out the use of this software herein. NormFinder employs a model-based variance estimation approach to identify genes suitable for normalization. In practice, it estimates both the intra- and intergroup variation and combines them into a stability value. This model-based approach ranks the top genes with minimal estimated inter- and intra-group variation. By contrast, the pairwise approach of geNorm selects two genes with the highest degree of expression profile similarity and the lowest intra-group variation. It is therefore not surprising that the two algorithms differed in their rankings of the best candidate genes. Nonetheless, regardless of such differences in the ranking order, the most unstable gene was almost always the same in all sample sets, as also observed in other studies [4], [9], [12].

In summary, this study is the first attempt to validate a set of candidate reference genes in different cultivars of sweetpotato for the normalization of gene expression analysis using RT-qPCR. Our results suggest that, for normalization, different suitable reference genes or combinations thereof should be selected based on the given experimental conditions and the particular cultivars. The results of the analysis of *IbLEA14* and *swpa2* expression emphasized the importance of validating reference genes to achieve accurate RT-qPCR results. These findings provide a foundation for the more accurate and widespread use of RT-qPCR in the analysis of gene expression in sweetpotato.

## Supporting Information

Figure S1 **Leaves, storage roots, and effects of stress conditions in four different sweetpotato cultivars.** (A) Leaves and storage roots of different colored sweetpotato cultivars. (B) Visible damage under cold, salt, drought and oxidative stress conditions. YM (Yulmi), SZM (Sinzami), Sinhwangmi (SHM), and WS (Whitestar).
(TIF)

Figure S2 **Melting curves of tested reference genes: For each reference gene melting curves were analyzed to verify the presence of a single product.** (A) *Actin*, (B) Cyclophilin, (C) *Glyceraldeide-3-phosphate dehydrogenase*, (D) *phospholipase D1α*, (E) *α -tubulin*, (F) *ADP-ribosylation factor*, (G) *cytochrome c oxidase subunit Vc*, (H) *histone H2B*, (I) *ribosomal protein L*, (J) *ubiquitin extension protein*.
(TIF)

Figure S3 **Pairwise variation (V) analysis of the candidate reference genes.** The pairwise variation (Vn/Vn +1) was analyzed between the normalization factors NF_n_ and NF_n+1_ by the geNorm software to determine the optimal number of reference genes required for RT-qPCR data normalization. *Arrow* indicates the optimal number of genes for normalization in each sample sets A) YM, B) SHM, C) SZM D) WS
(TIF)

Table S1 **The ten candidate genes for normalization and their expression stability values in various sample pools as calculated by geNorm or Normfinder.**
(DOCX)
