# Supplementary figures and images for: Stable Internal Reference Genes for the Normalization of Real-Time PCR in Different Sweetpotato Cultivars Subjected to Abiotic Stress Conditions
Source: PLoS One. 2012 Dec 12;7(12):e51502. doi: 10.1371/journal.pone.0051502 (PMC3520839; doi:10.1371/journal.pone.0051502)

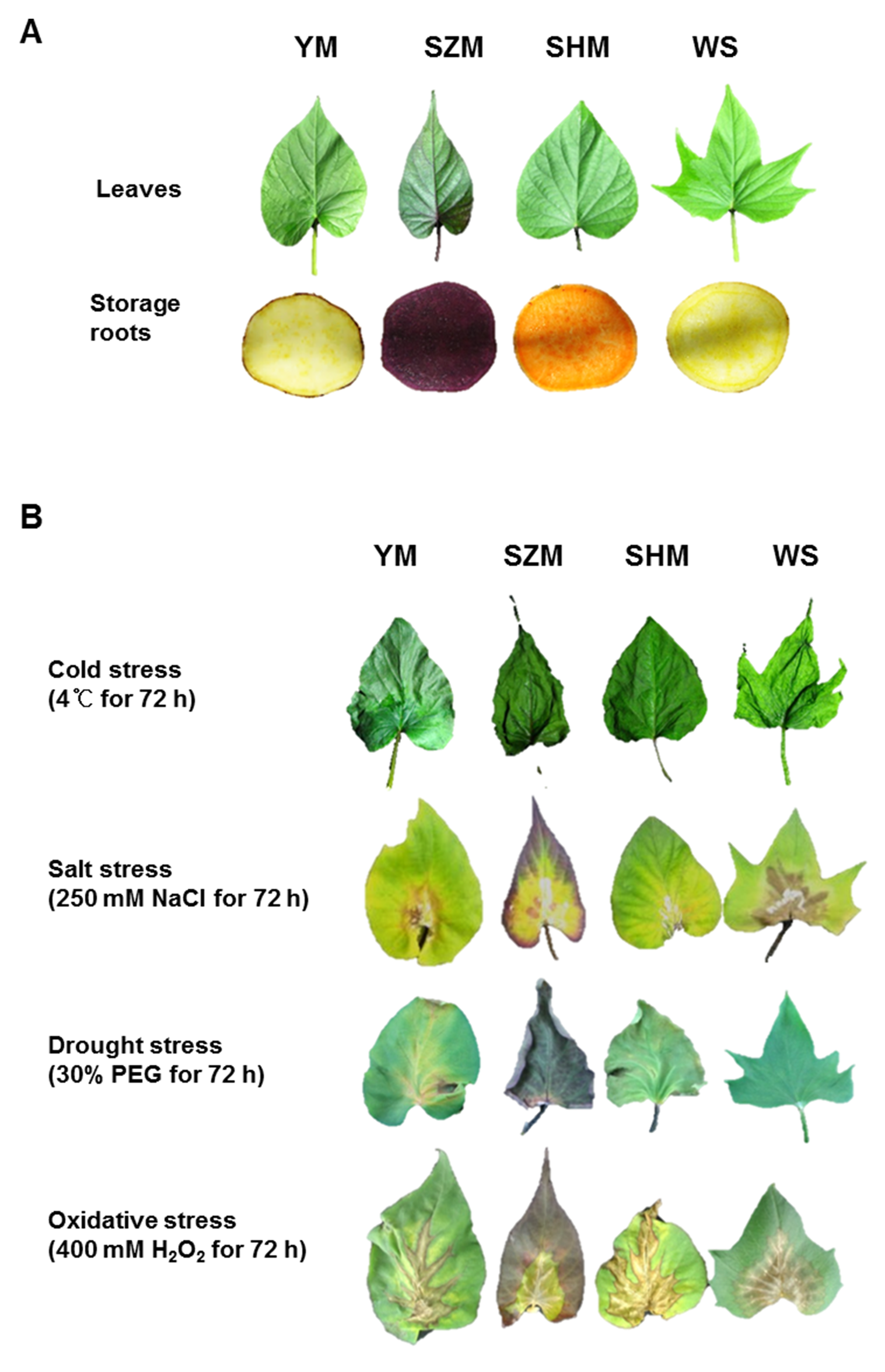

Supplement: Figure S1 — Leaves, storage roots, and effects of stress conditions in four different sweetpotato cultivars. (A) Leaves and storage roots of different colored sweetpotato cultivars. (B) Visible damage under cold, salt, drought and oxidative stress conditions. YM (Yulmi), SZM (Sinzami), Sinhwangmi (SHM), and WS (Whitestar). (TIF) [file pone.0051502.s001.tif]

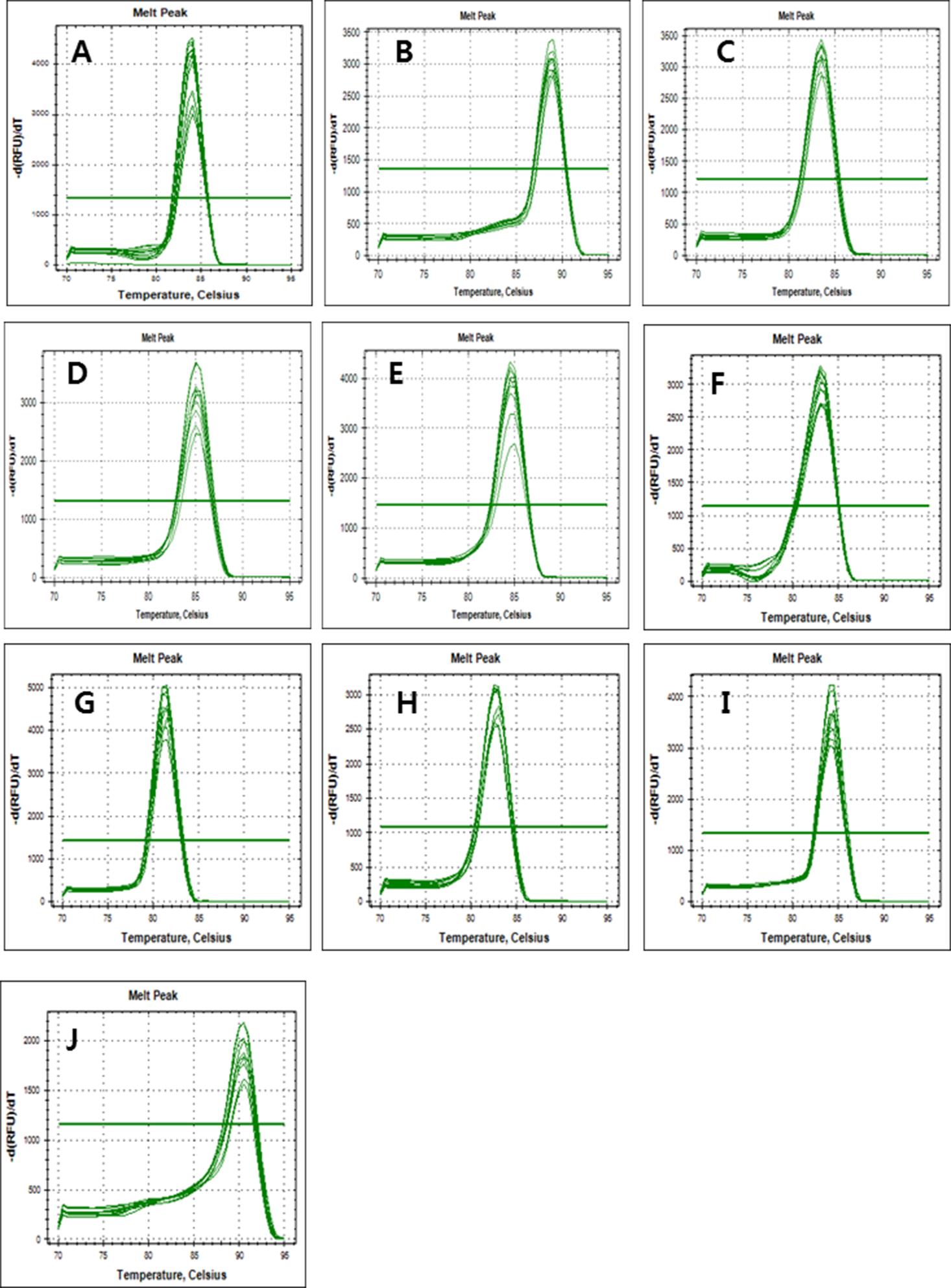

Supplement: Figure S2 — Melting curves of tested reference genes: For each reference gene melting curves were analyzed to verify the presence of a single product. (A) Actin, (B) Cyclophilin, (C) Glyceraldeide-3-phosphate dehydrogenase, (D) phospholipase D1α, (E) α -tubulin, (F) ADP-ribosylation factor, (G) cytochrome c oxidase subunit Vc, (H) histone H2B, (I) ribosomal protein L, (J) ubiquitin extension protein. (TIF) [file pone.0051502.s002.tif]

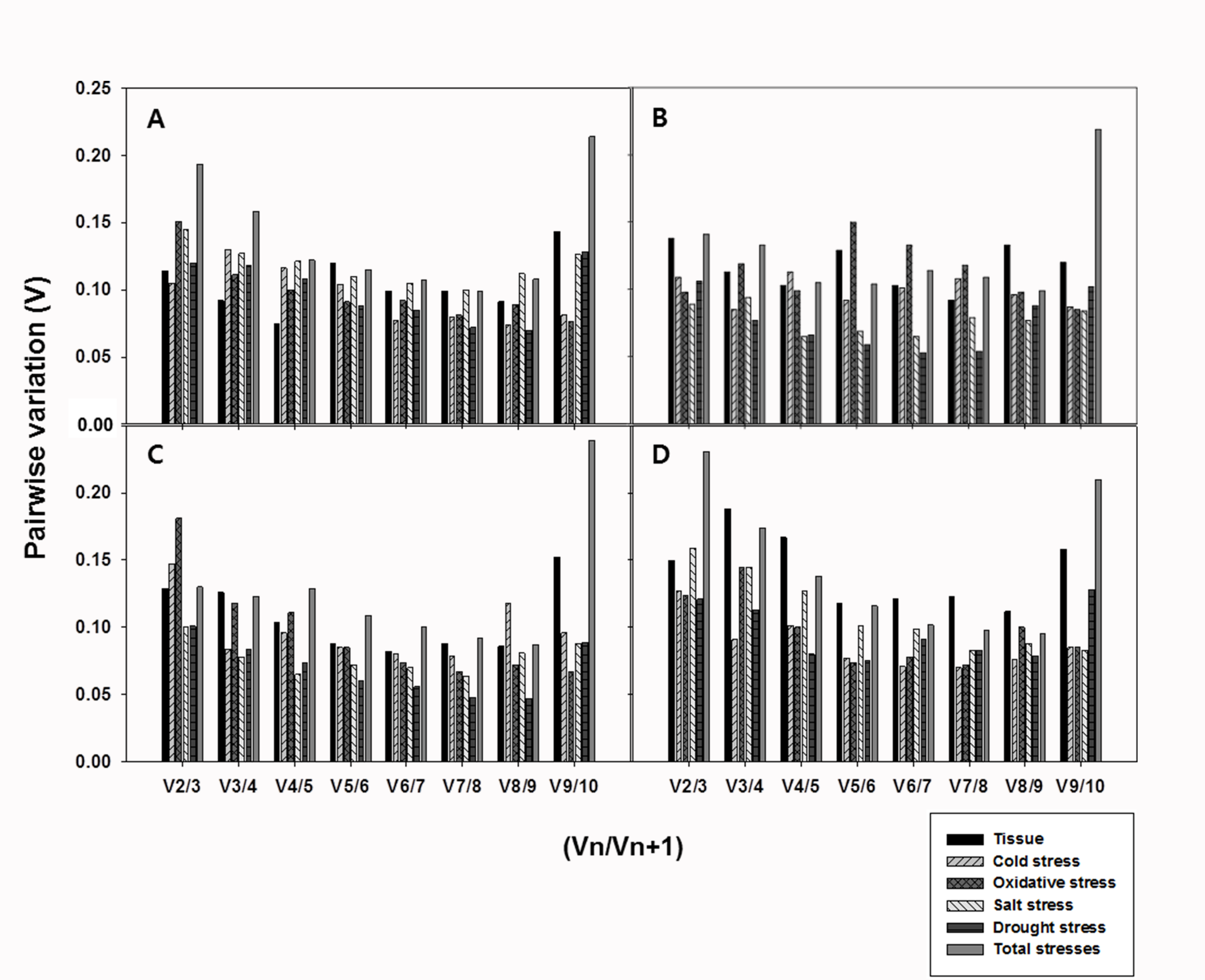

Supplement: Figure S3 — Pairwise variation (V) analysis of the candidate reference genes. The pairwise variation (Vn/Vn +1) was analyzed between the normalization factors NFn and NFn+1 by the geNorm software to determine the optimal number of reference genes required for RT-qPCR data normalization. Arrow indicates the optimal number of genes for normalization in each sample sets A) YM, B) SHM, C) SZM D) WS (TIF) [file pone.0051502.s003.tif]
