# Supplementary material for: Stable Internal Reference Genes for the Normalization of Real-Time PCR in Different Sweetpotato Cultivars Subjected to Abiotic Stress Conditions
Source: PLoS One. 2012 Dec 12;7(12):e51502. doi: 10.1371/journal.pone.0051502 (PMC3520839; doi:10.1371/journal.pone.0051502)
Supplement: Table S1 — The ten candidate genes for normalization and their expression stability values in various sample pools as calculated by geNorm or Normfinder. (DOCX) [file pone.0051502.s004.docx]

|  | YM | Different tissues | | Cold stress | | Oxidative stress | | Salt stress | | Drought stress | | All stresses | |
| --- | --- | --- | --- | --- | --- | --- | --- | --- | --- | --- | --- | --- | --- |
|  | Rank | Gene | Stability | Gene | Stability | Gene | Stability | Gene | Stability | Gene | Stability | Gene | Stability |
| g  e  N  o  r  m | 1 | *TUB* | 0.336 | *UBI* | 0.289 | *PLD* | 0.37 | *UBI* | 0.363 | *GAP* | 0.295 | *ARF* | 0.459 |
|  | 2 | *ACT* | 0.350 | *ARF* | 0.293 | *RPL* | 0.385 | *ARF* | 0.383 | *ARF* | 0.322 | *UBI* | 0.475 |
|  | 3 | *COX* | 0.359 | *GAP* | 0.309 | *H2B* | 0.415 | *PLD* | 0.408 | *PLD* | 0.341 | *COX* | 0.518 |
|  | 4 | *CYC* | 0.391 | *ACT* | 0.428 | *ARF* | 0.461 | *H2B* | 0.484 | *ACT* | 0.426 | *CYC* | 0.609 |
|  | 5 | *ARF* | 0.411 | *CYC* | 0.505 | *CYC* | 0.503 | *COX* | 0.558 | *UBI* | 0.491 | *H2B* | 0.652 |
|  | 6 | *PLD* | 0.529 | *PLD* | 0.571 | *GAP* | 0.535 | *CYC* | 0.617 | *H2B* | 0.533 | *GAP* | 0.7 |
|  | 7 | *RPL* | 0.588 | *RPL* | 0.586 | *UBI* | 0.587 | *GAP* | 0.679 | *COX* | 0.579 | *PLD* | 0.749 |
|  | 8 | *GAP* | 0.660 | *H2B* | 0.622 | *COX* | 0.626 | *TUB* | 0.734 | *CYC* | 0.601 | *TUB* | 0.793 |
|  | 9 | *UBI* | 0.721 | *COX* | 0.654 | *TUB* | 0.684 | *RPL* | 0.816 | *TUB* | 0.632 | *ACT* | 0.862 |
|  | 10 | *H2B* | 0.876 | *TUB* | 0.706 | *ACT* | 0.722 | *ACT* | 0.923 | *RPL* | 0.770 | *RPL* | 1.132 |
| N  o  r  m  f  i  n  d  e  r | 1 | *ARF* | 0.159 | *UBI* | 0.193 | *ARF* | 0.028 | *ARF* | 0.096 | *GAP* | 0.139 | *UBI* | 0.245 |
|  | 2 | *COX* | 0.313 | *ACT* | 0.195 | *H2B* | 0.206 | *UBI* | 0.144 | *PLD* | 0.180 | *ARF* | 0.258 |
|  | 3 | *CYC* | 0.323 | *CYC* | 0.300 | *PLD* | 0.285 | *PLD* | 0.239 | *ARF* | 0.026 | *H2B* | 0.279 |
|  | 4 | *PLD* | 0.338 | *ARF* | 0.308 | *RPL* | 0.314 | *H2B* | 0.305 | *UBI* | 0.302 | *CYC* | 0.316 |
|  | 5 | *ACT* | 0.367 | *PLD* | 0.323 | *CYC* | 0.341 | *TUB* | 0.413 | *ACT* | 0.315 | *PLD* | 0.349 |
|  | 6 | *RPL* | 0.388 | *GAP* | 0.327 | *UBI* | 0.343 | *GAP* | 0.505 | *H2B* | 0.326 | *TUB* | 0.371 |
|  | 7 | *GAP* | 0.436 | *RPL* | 0.331 | *GAP* | 0.445 | *COX* | 0.545 | *COX* | 0.402 | *GAP* | 0.391 |
|  | 8 | *TUB* | 0.469 | *H2B* | 0.458 | *COX* | 0.466 | *CYC* | 0.565 | *CYC* | 0.408 | *COX* | 0.438 |
|  | 9 | *UBI* | 0.471 | *COX* | 0.489 | *TUB* | 0.500 | *RPL* | 0.604 | *TUB* | 0.492 | *ACT* | 0.568 |
|  | 10 | *H2B* | 0.983 | *TUB* | 0.552 | *ACT* | 0.516 | *ACT* | 0.864 | *RPL* | 0.879 | *RPL* | 1.207 |

**Table S1. The ten candidate genes for normalization and their expression stability values in various sample pools as calculated by geNorm or Normfinder.**

|  | SHM | Different tissues | | Cold stress | | Oxidative stress | | Salt stress | | Drought stress | | All stresses | |
| --- | --- | --- | --- | --- | --- | --- | --- | --- | --- | --- | --- | --- | --- |
|  | Rank | Gene | Stability | Gene | Stability | Gene | Stability | Gene | Stability | Gene | Stability | Gene | Stability |
| g  e  N  o  r  m | 1 | *COX* | 0.386 | *H2B* | 0.314 | *COX* | 0.259 | *H2B* | 0.243 | *ARF* | 0.244 | *ARF* | 0.401 |
|  | 2 | *TUB* | 0.406 | *UBI* | 0.317 | *GAP* | 0.286 | *ARF* | 0.252 | *GAP* | 0.257 | *UBI* | 0.423 |
|  | 3 | *ARF* | 0.420 | *ARF* | 0.329 | *ARF* | 0.296 | *COX* | 0.264 | *UBI* | 0.281 | *COX* | 0.436 |
|  | 4 | *CYC* | 0.459 | *CYC* | 0.357 | *CYC* | 0.397 | *UBI* | 0.332 | *H2B* | 0.316 | *H2B* | 0.51 |
|  | 5 | *ACT* | 0.506 | *GAP* | 0.456 | *UBI* | 0.459 | *PLD* | 0.350 | *PLD* | 0.340 | *CYC* | 0.551 |
|  | 6 | *PLD* | 0.615 | *COX* | 0.513 | *H2B* | 0.618 | *RPL* | 0.390 | *COX* | 0.365 | *GAP* | 0.606 |
|  | 7 | *RPL* | 0.659 | *TUB* | 0.586 | *ACT* | 0.726 | *GAP* | 0.425 | *CYC* | 0.385 | *PLD* | 0.686 |
|  | 8 | *GAP* | 0.708 | *ACT* | 0.673 | *PLD* | 0.802 | *CYC* | 0.489 | *ACT* | 0.411 | *ACT* | 0.759 |
|  | 9 | *UBI* | 0.839 | *PLD* | 0.735 | *RPL* | 0.837 | *TUB* | 0.550 | *TUB* | 0.504 | *TUB* | 0.819 |
|  | 10 | *H2B* | 0.937 | *RPL* | 0.776 | *TUB* | 0.875 | *ACT* | 0.622 | *RPL* | 0.618 | *RPL* | 1.106 |
| N  o  r  m  f  i  n  d  e  r | 1 | *ARF* | 0.061 | *ARF* | 0.152 | *ARF* | 0.258 | *ARF* | 0.075 | *UBI* | 0.119 | *H2B* | 0.241 |
|  | 2 | *COX* | 0.295 | *H2B* | 0.162 | *H2B* | 0.293 | *H2B* | 0.096 | *H2B* | 0.127 | *CYC* | 0.340 |
|  | 3 | *ACT* | 0.363 | *CYC* | 0.186 | *CYC* | 0.378 | *PLD* | 0.186 | *ARF* | 0.128 | *PLD* | 0.343 |
|  | 4 | *TUB* | 0.365 | *UBI* | 0.221 | *GAP* | 0.406 | *COX* | 0.196 | *GAP* | 0.163 | *UBI* | 0.349 |
|  | 5 | *CYC* | 0.407 | *TUB* | 0.425 | *COX* | 0.415 | *UBI* | 0.212 | *PLD* | 0.226 | *ARF* | 0.353 |
|  | 6 | *GAP* | 0.425 | *ACT* | 0.449 | *ACT* | 0.463 | *RPL* | 0.296 | *ACT* | 0.226 | *GAP* | 0.422 |
|  | 7 | *PLD* | 0.458 | *GAP* | 0.502 | *PLD* | 0.532 | *GAP* | 0.300 | *COX* | 0.241 | *COX* | 0.449 |
|  | 8 | *RPL* | 0.514 | *PLD* | 0.510 | *TUB* | 0.566 | *TUB* | 0.465 | *CYC* | 0.310 | *TUB* | 0.456 |
|  | 9 | *UBI* | 0.763 | *COX* | 0.548 | *UBI* | 0.580 | *CYC* | 0.468 | *TUB* | 0.556 | *ACT* | 0.534 |
|  | 10 | *H2B* | 0.819 | *RPL* | 0.588 | *RPL* | 0.582 | *ACT* | 0.574 | *RPL* | 0.704 | *RPL* | 1.348 |

|  | SZM | Different tissues | | Cold stress | | Oxidative stress | | Salt stress | | Drought stress | | All stresses | |
| --- | --- | --- | --- | --- | --- | --- | --- | --- | --- | --- | --- | --- | --- |
|  | Rank | Gene | Stability | Gene | Stability | Gene | Stability | Gene | Stability | Gene | Stability | Gene | Stability |
| g  e  N  o  r  m | 1 | *PLD* | 0.223 | *UBI* | 0.273 | *PLD* | 0.299 | *COX* | 0.304 | *TUB* | 0.255 | *ARF* | 0.424 |
|  | 2 | *RPL* | 0.226 | *ARF* | 0.276 | *RPL* | 0.301 | *PLD* | 0.313 | *UBI* | 0.256 | *UBI* | 0.428 |
|  | 3 | *GAP* | 0.280 | *H2B* | 0.332 | *ARF* | 0.382 | *ARF* | 0.319 | *H2B* | 0.276 | *COX* | 0.432 |
|  | 4 | *ARF* | 0.407 | *CYC* | 0.360 | *H2B* | 0.458 | *UBI* | 0.340 | *ACT* | 0.323 | *H2B* | 0.489 |
|  | 5 | *ACT* | 0.474 | *COX* | 0.418 | *ACT* | 0.522 | *ACT* | 0.357 | *COX* | 0.361 | *CYC* | 0.578 |
|  | 6 | *COX* | 0.512 | *GAP* | 0.470 | *TUB* | 0.538 | *H2B* | 0.398 | *GAP* | 0.381 | *PLD* | 0.634 |
|  | 7 | *CYC* | 0.554 | *TUB* | 0.517 | *CYC* | 0.563 | *CYC* | 0.439 | *ARF* | 0.405 | *GAP* | 0.682 |
|  | 8 | *TUB* | 0.613 | *ACT* | 0.565 | *UBI* | 0.582 | *GAP* | 0.476 | *CYC* | 0.419 | *ACT* | 0.728 |
|  | 9 | *UBI* | 0.669 | *PLD* | 0.685 | *COX* | 0.616 | *RPL* | 0.545 | *PLD* | 0.438 | *TUB* | 0.77 |
|  | 10 | *H2B* | 0.849 | *RPL* | 0.748 | *GAP* | 0.65 | *TUB* | 0.625 | *RPL* | 0.535 | *RPL* | 1.103 |
| N  o  r  m  f  i  n  d  e  r | 1 | *GAP* | 0.134 | *H2B* | 0.089 | *ARF* | 0.046 | *COX* | 0.088 | *GAP* | 0.137 | *ARF* | 0.168 |
|  | 2 | *ARF* | 0.173 | *CYC* | 0.115 | *CYC* | 0.261 | *ARF* | 0.104 | *ACT* | 0.173 | *H2B* | 0.239 |
|  | 3 | *PLD* | 0.255 | *ARF* | 0.248 | *H2B* | 0.271 | *H2B* | 0.161 | *H2B* | 0.182 | *PLD* | 0.269 |
|  | 4 | *RPL* | 0.290 | *UBI* | 0.265 | *UBI* | 0.311 | *PLD* | 0.217 | *ARF* | 0.202 | *UBI* | 0.282 |
|  | 5 | *CYC* | 0.306 | *ACT* | 0.275 | *PLD* | 0.319 | *ACT* | 0.245 | *COX* | 0.216 | *COX* | 0.328 |
|  | 6 | *ACT* | 0.338 | *TUB* | 0.398 | *RPL* | 0.337 | *UBI* | 0.270 | *PLD* | 0.229 | *CYC* | 0.403 |
|  | 7 | *COX* | 0.340 | *GAP* | 0.441 | *ACT* | 0.363 | *GAP* | 0.329 | *CYC* | 0.240 | *ACT* | 0.485 |
|  | 8 | *UBI* | 0.451 | *COX* | 0.485 | *TUB* | 0.376 | *CYC* | 0.334 | *UBI* | 0.246 | *TUB* | 0.500 |
|  | 9 | *TUB* | 0.615 | *PLD* | 0.645 | *COX* | 0.424 | *RPL* | 0.518 | *TUB* | 0.306 | *GAP* | 0.595 |
|  | 10 | *H2B* | 1.048 | *RPL* | 0.653 | *GAP* | 0.449 | *TUB* | 0.602 | *RPL* | 0.613 | *RPL* | 1.398 |

|  | WS | Different tissues | | Cold stress | | Oxidative stress | | Salt stress | | Drought stress | | All stresses | |
| --- | --- | --- | --- | --- | --- | --- | --- | --- | --- | --- | --- | --- | --- |
|  | Rank | Gene | Stability | Gene | Stability | Gene | Stability | Gene | Stability | Gene | Stability | Gene | Stability |
| g  e  N  o  r  m | 1 | *COX* | 0.377 | *PLD* | 0.244 | *ARF* | 0.296 | *ARF* | 0.415 | *COX* | 0.371 | *COX* | 0.681 |
|  | 2 | *UBI* | 0.395 | *RPL* | 0.246 | *COX* | 0.304 | *COX* | 0.453 | *H2B* | 0.379 | *UBI* | 0.694 |
|  | 3 | *ARF* | 0.421 | *H2B* | 0.292 | *GAP* | 0.332 | *UBI* | 0.472 | *UBI* | 0.387 | *H2B* | 0.716 |
|  | 4 | *TUB* | 0.593 | *CYC* | 0.342 | *PLD* | 0.469 | *H2B* | 0.551 | *CYC* | 0.445 | *ARF* | 0.761 |
|  | 5 | *PLD* | 0.720 | *ARF* | 0.421 | *RPL* | 0.496 | *GAP* | 0.614 | *PLD* | 0.459 | *CYC* | 0.788 |
|  | 6 | *RPL* | 0.741 | *ACT* | 0.460 | *CYC* | 0.515 | *RPL* | 0.649 | *ACT* | 0.485 | *PLD* | 0.807 |
|  | 7 | *CYC* | 0.794 | *UBI* | 0.490 | *H2B* | 0.552 | *ACT* | 0.695 | *TUB* | 0.543 | *GAP* | 0.828 |
|  | 8 | *ACT* | 0.874 | *TUB* | 0.528 | *ACT* | 0.583 | *PLD* | 0.722 | *GAP* | 0.593 | *ACT* | 0.859 |
|  | 9 | *GAP* | 0.941 | *GAP* | 0.578 | *UBI* | 0.665 | *TUB* | 0.763 | *RPL* | 0.639 | *TUB* | 0.895 |
|  | 10 | *H2B* | 1.091 | *COX* | 0.647 | *TUB* | 0.724 | *CYC* | 0.801 | *ARF* | 0.780 | *RPL* | 1.153 |
| N  o  r  m  f  i  n  d  e  r | 1 | *ARF* | 0.122 | *H2B* | 0.153 | *ARF* | 0.159 | *ARF* | 0.079 | *PLD* | 0.169 | *PLD* | 0.255 |
|  | 2 | *UBI* | 0.340 | *ARF* | 0.176 | *GAP* | 0.204 | *H2B* | 0.268 | *H2B* | 0.190 | *H2B* | 0.278 |
|  | 3 | *TUB* | 0.344 | *UBI* | 0.190 | *COX* | 0.238 | *COX* | 0.295 | *CYC* | 0.204 | *ARF* | 0.315 |
|  | 4 | *COX* | 0.388 | *CYC* | 0.202 | *H2B* | 0.323 | *UBI* | 0.316 | *COX* | 0.232 | *UBI* | 0.374 |
|  | 5 | *PLD* | 0.502 | *RPL* | 0.325 | *PLD* | 0.338 | *GAP* | 0.369 | *UBI* | 0.232 | *GAP* | 0.412 |
|  | 6 | *CYC* | 0.526 | *PLD* | 0.338 | *CYC* | 0.353 | *PLD* | 0.458 | *ACT* | 0.268 | *CYC* | 0.417 |
|  | 7 | *RPL* | 0.550 | *TUB* | 0.352 | *RPL* | 0.374 | *RPL* | 0.464 | *GAP* | 0.466 | *TUB* | 0.449 |
|  | 8 | *GAP* | 0.621 | *ACT* | 0.368 | *ACT* | 0.374 | *ACT* | 0.502 | *RPL* | 0.476 | *ACT* | 0.480 |
|  | 9 | *ACT* | 0.721 | *GAP* | 0.433 | *UBI* | 0.578 | *TUB* | 0.509 | *TUB* | 0.484 | *COX* | 0.494 |
|  | 10 | *H2B* | 1.080 | *COX* | 0.582 | *TUB* | 0.578 | *CYC* | 0.561 | *ARF* | 0.882 | *RPL* | 1.315 |

|  | All | Different tissues | | Cold stress | | Oxidative stress | | Salt stress | | Drought stress | | All stresses | | Total | |
| --- | --- | --- | --- | --- | --- | --- | --- | --- | --- | --- | --- | --- | --- | --- | --- |
|  | Rank | Gene | Stability | Gene | Stability | Gene | Stability | Gene | Stability | Gene | Stability | Gene | Stability | Gene | Stability |
| g  e  N  o  r  m | 1 | *PLD* | 0.419 | *UBI* | 0.528 | *RPL* | 0.422 | *ARF* | 0.421 | *COX* | 0.49 | *UBI* | 0.571 | *ARF* | 0.669 |
|  | 2 | *RPL* | 0.432 | *ARF* | 0.529 | *PLD* | 0.423 | *COX* | 0.455 | *UBI* | 0.496 | *COX* | 0.585 | *UBI* | 0.694 |
|  | 3 | *ARF* | 0.529 | *H2B* | 0.597 | *H2B* | 0.487 | *UBI* | 0.469 | *PLD* | 0.507 | *ARF* | 0.591 | *COX* | 0.714 |
|  | 4 | *COX* | 0.615 | *ACT* | 0.712 | *ARF* | 0.639 | *PLD* | 0.519 | *CYC* | 0.538 | *H2B* | 0.693 | *GAP* | 0.819 |
|  | 5 | *ACT* | 0.669 | *CYC* | 0.749 | *GAP* | 0.691 | *H2B* | 0.588 | *GAP* | 0.559 | *CYC* | 0.738 | *PLD* | 0.862 |
|  | 6 | *TUB* | 0.691 | *PLD* | 0.771 | *COX* | 0.722 | *CYC* | 0.648 | *H2B* | 0.600 | *PLD* | 0.783 | *CYC* | 0.898 |
|  | 7 | *CYC* | 0.713 | *TUB* | 0.803 | *CYC* | 0.752 | *GAP* | 0.715 | *TUB* | 0.640 | *GAP* | 0.815 | *TUB* | 0.946 |
|  | 8 | *GAP* | 0.774 | *GAP* | 0.832 | *UBI* | 0.772 | *RPL* | 0.761 | *ACT* | 0.664 | *TUB* | 0.868 | *ACT* | 0.986 |
|  | 9 | *UBI* | 0.824 | *RPL* | 0.872 | *ACT* | 0.791 | *TUB* | 0.803 | *ARF* | 0.703 | *ACT* | 0.908 | *H2B* | 1.027 |
|  | 10 | *H2B* | 0.951 | *COX* | 0.909 | *TUB* | 0.81 | *ACT* | 0.852 | *RPL* | 0.777 | *RPL* | 1.18 | *RPL* | 1.262 |
| N  o  r  m  f  i  n  d  e  r | 1 | *ARF* | 0.049 | *H2B* | 0.212 | *ARF* | 0.200 | *ARF* | 0.137 | *PLD* | 0.159 | *H2B* | 0.192 | *H2B* | 0.192 |
|  | 2 | *COX* | 0.126 | *ARF* | 0.259 | *RPL* | 0.230 | *UBI* | 0.178 | *UBI* | 0.165 | *PLD* | 0.192 | *PLD* | 0.192 |
|  | 3 | *PLD* | 0.147 | *PLD* | 0.287 | *COX* | 0.248 | *COX* | 0.209 | *CYC* | 0.201 | *UBI* | 0.213 | *UBI* | 0.213 |
|  | 4 | *CYC* | 0.148 | *UBI* | 0.310 | *H2B* | 0.249 | *PLD* | 0.226 | *TUB* | 0.225 | *ARF* | 0.213 | *ARF* | 0.213 |
|  | 5 | *GAP* | 0.153 | *ACT* | 0.318 | *UBI* | 0.250 | *RPL* | 0.277 | *ARF* | 0.233 | *CYC* | 0.259 | *CYC* | 0.259 |
|  | 6 | *RPL* | 0.165 | *CYC* | 0.374 | *ACT* | 0.253 | *CYC* | 0.313 | *COX* | 0.244 | *TUB* | 0.298 | *TUB* | 0.298 |
|  | 7 | *TUB* | 0.169 | *TUB* | 0.380 | *PLD* | 0.254 | *TUB* | 0.321 | *GAP* | 0.255 | *GAP* | 0.310 | *GAP* | 0.310 |
|  | 8 | *ACT* | 0.169 | *RPL* | 0.413 | *TUB* | 0.257 | *H2B* | 0.322 | *H2B* | 0.274 | *COX* | 0.321 | *COX* | 0.321 |
|  | 9 | *UBI* | 0.191 | *GAP* | 0.422 | *CYC* | 0.281 | *ACT* | 0.343 | *RPL* | 0.310 | *ACT* | 0.434 | *ACT* | 0.434 |
|  | 10 | *H2B* | 0.371 | *COX* | 0.449 | *GAP* | 0.282 | *GAP* | 0.416 | *ACT* | 0.324 | *RPL* | 1.152 | *RPL* | 1.152 |
